# Measuring the Delivery of Complex Interventions through Electronic Medical Records: Challenges and Lessons Learned

**DOI:** 10.5334/egems.230

**Published:** 2018-05-25

**Authors:** Beth Prusaczyk, Vanessa Fabbre, Christopher R. Carpenter, Enola Proctor

**Affiliations:** 1 Vanderbilt University Medical Center, US; 2 Washington University in St. Louis, US

**Keywords:** Electronic Health Records, Implementation Science, Health Services Research, Delivery of Health Care, Quality Improvement

## Abstract

**Background:**

Health services and implementation researchers often seek to capture the implementation process of complex interventions yet explicit guidance on how to capture this process is limited. Medical record review is a commonly used methodology, especially when used as a proxy for provider behavior, with recognized benefits and limitations. The purpose of this study was to test the feasibility of chart review to measure implementation and offer recommendations for future researchers using this method to capture the implementation process.

**Methods:**

Grounded in qualitative research methods, we measured the implementation of a transitional care intervention for older adults with dementia being discharged from the hospital. We adapted the operationalization of the intervention’s components to suit chart review methods, sought input from hospital providers before and after data collection, and assessed the agreement between the results of our chart review and provider-report.

**Findings:**

We believe chart review can be used effectively as a method for capturing the implementation process and provide future researchers with a list of recommendations based on our experience including understanding the nuance between data extraction versus data abstraction, allowing for large amounts of data not pre-specified in the data collection instrument to be collected, and purposefully and iteratively engaging the providers who are entering data into the chart.

**Major Themes:**

Measuring the implementation of complex interventions is a cornerstone in health services research and with the relative convenience and low costs of using chart data, we believe with more use and refinement this methodology could emerge as a valuable and widely used method in the field.

## Background

The Medical Research Council’s framework for the development and evaluation of complex interventions emphasizes the importance of evaluating the process of implementing complex interventions [1]. However, the framework does not discuss *how* to evaluate the process nor does it discuss the challenges researchers and evaluators might encounter when measuring the process of implementing complex interventions. Explicit methods for reporting the implementation of complex interventions are needed to more fully attend to the potential biases, content validity, reliability, and reproducibility of findings across settings and between medical specialties [2]. We aimed to contribute methodological guidance in this area by testing the feasibility of using a common methodology (medical chart review) to evaluate the process of implementing a complex intervention (transitional care).

Medical chart review is a type of methodology “in which prerecorded, patient-centered data are used to answer one or more research questions [3].” Despite known challenges to using medical chart review, including evidence of poor documentation by providers and poor sensitivity and specificity of results, it remains a commonly used methodology in clinical and health services research [4,5,6,7,8]. The availability of medical charts, the ability to collect data from a large sample, and in certain circumstances the relatively low cost are just a few reasons researchers may choose to use this methodology [8,9,10,11]. It is not clear how often or for what purpose this methodology is used in implementation research but a review of valid proxy measures of clinician behavior reported that of the 15 included studies nine (60 percent) used medical chart review as a proxy to direct observation [12], suggesting that chart review is a frequently used method in the field. And while there is extensive guidance for researchers seeking to use this method in other fields [3,4,7,13,14], no methodological guidance has been put forth for using chart review techniques specifically for implementation research.

Transitional care is a complex intervention designed to ensure the coordination and continuity of health care as patients transition between different locations or levels of care [20] and often includes actions such as early assessment of needs for follow-up resources, medication reconciliation, discharge planning, providing education and support to the patient and caregivers, and coordination among health care professionals [21,22,23,24,25,26].

We assessed the extent to which components of the intervention are identifiable in charts, by calculating the time and resources required for chart extraction, and by assessing the agreement between data from chart review and provider report. We also summarize our experiences including the steps we used and the challenges we faced in order to guide future researchers.

## Methods

As stated earlier, the purpose of this paper is to contribute to the literature on how to measure the implementation of complex interventions. We believe our study on the implementation of transitional care for older adults with dementia being discharged from the hospital can serve as a case study through which methodological insights in this area can be gained. Below we present a description of the study and our methods. In the Findings section we then discuss the accuracy and usefulness of our methods within the context of the implementation of complex interventions.

### Description of Case Study

Numerous specific *transitional care interventions* exist with different combinations of transitional care actions [27,28,29,30,31,32,33,34] and many are already being used, though not routinely, in clinical practice. In an effort to catalog and prioritize all of the transitional care actions utilized in the interventions, Burke et al. created the Ideal Transitions in Care framework [35]. The Ideal Transitions in Care framework conceptualizes the ideal transitional care intervention by identifying ten actions that support patients during a care transition. To remain consistent with the authors’ terminology we will continue to refer to the Ideal Transitions in Care as a framework, but it can also be thought of as a checklist of what a patient should ideally receive during a care transition. The Ideal Transitions in Care framework meets the Medical Research Council’s definition of a complex intervention because it encompasses numerous interacting components, involves a number of groups or organizational levels, and has a number of outcomes [1]. This, along with its “checklist”-like format, made it an ideal intervention for the purpose of this study.

We believe chart review was an appropriate method for our research study because we were able to specifically capture the implementation process including what was implemented, by whom, how, where, and when [15]. When appropriate and feasible, we followed existing guidelines for chart review and recommend other researchers do the same [3,4,7,13,14,16]. However, because we sought to identify patterns of implementation and generate hypotheses, we also followed appropriate standards and methods for a novel, qualitative, and exploratory study. How we followed these standards in our study is discussed in more detail below but resources exist for readers looking for more information on the qualitative methods underpinning our study [17,18,19].

### Sampling Procedures

All study data were collected from a large, urban teaching hospital in St. Louis, Missouri. We did not restrict our sample based on certain diagnoses or symptoms or to patients on a specific unit or floor of the hospital. The only criteria used to define our sample were age at discharge, date of discharge, and length of stay. In order to be included in our study population patients had to be ≥70 years old at the time of hospital discharge with a discharge date between January 1, 2015, and December 31, 2015, and had to have an inpatient length of stay ≥1 day. We chose a one-year time frame in an effort to minimize any institutional changes that may have occurred at the hospital that would have influenced the transitional care provided.

We stratified our sample based on two criteria: whether the patient had a dementia diagnosis and whether the patient received surgery during their inpatient stay. With our study population and strata determined, we reviewed a random sample of charts until topical saturation was reached [18,19]. All charts were reviewed by a single coder (BP).

### Development of Data Abstraction Tool

Creating the data collection instrument involved multiple sub-steps and their importance cannot be overstated. First, because the definitions from the Ideal Transitions in Care were not intended for chart review purposes, some had to be adapted. For example, “Monitoring and Managing Symptoms After Discharge” is an action that takes place after the patient leaves the hospital and thus would not be captured in the inpatient medical record. Therefore, we defined this action as whether or not there was evidence in the record that the patient or caregiver received information in the hospital about how to monitor and manage symptoms after discharge. This process was not specific to implementation research and was driven instead by the need to adapt the framework to the chart review methodology. As such, we anticipate all researchers conducting a chart review will go through this adaptation process if they are using a framework or theory to guide their chart review and we recommend they follow existing recommendations for creating a data collection instrument [3,4,7,8,9]. A full description of the adaptation and operationalization of these variables can be found in Table 1.

**Table 1:** Operationalization of Ideal Transition in Care framework for chart review.

Ideal Transition in Care Framework Definition	Operationalization

*Discharge Planning*: Planning ahead for hospital discharge while the patient is still being treated in the hospital. Includes collaborating with the outpatient provider and taking the patient and caregiver’s preferences for appointment scheduling into account.	This will be any indication of discharge planning either by completed Discharge Summary form or any mention of “discharge plan/planning” in the free-text documentation.
*Complete Communication of Information*: The content that should be included in the discharge summaries and other means of information transfer from hospital to post-discharge care.	At a minimum, the following information coded as sub-actions should be included in the discharge summary or documentation: 1) Primary and secondary diagnoses, 2) discharge medications, 3) results of procedures, 4) follow-up needs, and 5) Pending test results.
*Availability, Timeliness, Clarity, and Organization of Information*: The availability, timeliness, clarity, and organization of the information above ensure post-discharge providers can access and quickly understand the information before assuming care of the patient.	All information will be considered *available* since it was by nature available in the medical record. The information will be considered *timely* if there is any indication the Discharge Summary form was provided to the PCP prior to discharge or first scheduled follow-up appointment. The *clarity and organization* of the information will be coded if the Discharge Summary or note contains sub-headings or bullet-points.
*Medication Safety*: 1) Taking an accurate medication history, 2) reconciling changes throughout hospitalization, and 3) communicating the reconciled medication regimen to patients and providers across transitions of care.	One of the three sub-actions indicated either by completed Discharge Medication Report or mention of “medication history” or “medication reconciliation” or mention of discussing medications with patient or PCP in free-text documentation.
*Patient Education & Promotion of Self-Management*: Teaching patients and their caregivers about 1) the main hospital diagnoses and instructions for self-care, including 2) medication changes, 3) appointments, and 4) whom to contact if issues arise. Confirming comprehension of instructions through 5) assessment of delirium and dementia and 6) teach-back, and 7) providing educational materials that are appropriate to the patient and caregiver’s level of health literacy and preferred language are important.	One of these seven sub-actions indicated by either completed form or mention in the free-text documentation.
*Social and Community Supports*: Enlisting the help of these supports is crucial for assisting patients with household activities, meals, and other necessities during recovery.	Any indication – by either completed form or mention in documentation – of contacting, enlisting, or utilizing community and social supports.
*Advance Care Planning*: May begin in hospital or outpatient setting and involves 1) establishing goals of care and 2) health care proxies, as well as 3) engaging with palliative or hospice care if appropriate.	One of these sub-actions indicated by either completed form or mention in the free-text documentation.
*Coordinating Care Among Team Members*: Synchronizing efforts across settings and providers is vital as they coordinate information, assessments, and plans as a team.	This will be any indication of communication between the hospital and any outside providers either by completed form or mention in the free-text documentation.
*Monitoring and Managing Symptoms after Discharge:* Monitoring for new or worsening symptoms, medication side effects, discrepancies, or non-adherence, and other self-management challenges.	Any indication the patient/caregiver was educated on any one of these sub-actions: 1) Post-discharge symptoms, 2) Post-discharge medication side effects, 3) Medication regimen, 4) Inquired about other self-management challenges.
*Outpatient Follow-up:* Appropriate and prompt post-discharge appointments with providers who have a longitudinal relationship with the patient.	This will be any indication of scheduled follow-up appointments with either the patient’s PCP or a specialty provider by completed form or mention in free-text documentation.

We also collected, when available, information on the intervention’s implementation features, such as who provided each action of the intervention, when the action was provided, and details on the action (i.e., what specific community or social supports were arranged) [15]. This step was specific to implementation research and we believe is partially what separates a chart review study for implementation research apart from other types of research. These details help to map the implementation process and as we will discuss below are often readily available in the charts.

Next, we sought input from hospital providers on our operationalization of transitional care and our data collection instrument. We chose to interview a small, select sample of hospital providers to identify additional transitional care actions routinely provided to patients not represented in the framework *and* to learn where providers routinely documented their actions, if they were documented at all. The structured interview guide is available [see Supplemental File 1]. We interviewed a total of nine providers including a physician, registered nurse, case manager, and pharmacist. We also interviewed two advanced practice nurses and two social workers because these are the two types of providers most often used to deliver transitional care interventions in the literature [27,31,36,37], and we wanted to gain multiple perspectives from these roles. For these two roles we interviewed one provider from a surgical unit and one from a non-surgical unit because we anticipated there might be differences in the transitional care needs of patients who had received surgery compared to patients who had not. Lastly, we interviewed one case manager from the emergency department to ensure we captured the perspective of providers in this unique setting. We determined that one type of each provider with additional providers from the types most common in the literature was an appropriate sample for our purpose. A single interviewer (BP) conducted all interviews.

These providers did not identify any additional transitional care activities they routinely provide to patients outside of those listed in the framework. However, we did learn valuable information about when and where providers document their actions. This information was useful not only for reviewing the charts but also for interpreting the results. A more detailed discussion of the usefulness of this information follows in the Results section.

Lastly, with our data collection instrument prepared, we pilot tested it on a random sample of 20 charts and made revisions accordingly. This pilot-testing process is not novel to our study and is recommended for all chart review studies [3,4,7,13]. All charts reviewed in the pilot test were done by a single coder (BP) and the revision process was done with the coder and another lead research team member (EP). We pilot tested the data collection instrument until the coder felt comfortable with the instrument and felt that no new information was being revealed in the charts that would specifically affect whether or not the instrument needed to be adapted.

### Chart Review Procedures

The data collection process in our study mirrored data collection for other chart review studies in that a coder accessed the electronic medical record (EMR) and reviewed the chart while simultaneously extracting and abstracting data from the chart and entering it into the electronic data collection form. A discussion of the lessons we learned while reviewing the charts is presented below in the next section but the literal process of data collection did not differ considerably from chart reviews for non-implementation research studies. On average it took 44 minutes to review one chart with a range of 34 to 97 minutes. We reached topical saturation with a sample size of 210 patient charts. Saturation was determined through discussion by authors BP, EP, and VF.

### Validity Check

After data collection and analysis, we sought to assess the face validity of the chart review methodology for measuring a complex intervention including its implementation features. We did this by interviewing the same hospital providers we interviewed prior to data collection. Providers were asked a number of open-ended questions to elicit their thoughts on the results, including “What are your initial thoughts about the results?” and “Did you find anything surprising about the results?” Every provider was asked specifically, “Do these results match what you see in your day-to-day practice?” Their responses allowed us to assess the face validity of the data obtained in the chart review and add context to the results. A single interviewer (BP) conducted all interviews.

Through these interviews we were not only able to qualitatively assess the validity of the chart review results but also quantify the agreement between what providers said they routinely provide and document and what was found in the chart. It is important to state that we are not making a judgment as to whether provider-report or chart documentation is more accurate or valid and we recognize that what truly happens in practice likely lies somewhere in between what providers say and what is documented in the chart. However, quantifying the *agreement* between provider-report and chart review is useful in the case of implementation research and will be discussed in more detail later.

## Findings

We believe this methodology was effective at measuring the complex intervention of transitional care based on our confidence in reaching saturation and the post-analysis provider interviews. For example, we found that some transitional care actions, such as discharge planning, were delivered to 100 percent of patients. The fact that we were able to find evidence of this transitional care action for all patients suggests that chart review was accurately capturing this process and our methodology was appropriate.

Providers confirmed the face validity of the chart review data for a majority of transitional care actions. For example, the chart review data suggested that registered nurses were most often the ones providing education to patients while social workers were almost always involved in facilitating a patient’s discharge. During their interviews, both registered nurses and social workers confirmed these roles. Furthermore, we not only asked providers to confirm their own roles but also the roles of other providers. For instance, in the previous example we asked social workers if nurses are the ones primarily providing education to patients and social workers confirmed that nurses are. Providers partially confirmed all but one of the remaining actions – meaning they confirmed what the chart review data revealed but also said there were additional transitional care actions and implementation features that were not captured in the chart review data. For example, a nurse practitioner said that they, along with social workers, were often the ones describing hospice and palliative care to patients and their families. This was not found in the chart data but the nurse practitioner said they do not often document this activity.

Only one transitional care action – complete communication of information – did not need a validity check by providers because it was measured unequivocally by the presence or absence of key pieces of information in the discharge summary and there was no uncertainty about the validity of these data.

Our experience suggests that chart review methodology is an effective way to measure a complex intervention including its implementation features. However, there are many challenges to using this methodology for this purpose. Below we discuss these challenges and the lessons we learned through this study below (summarized in Table 2) and highlight key implications for future researchers who use this methodology. We end with a list of recommendations for conducting and reporting chart review studies for implementation research.

**Table 2:** Challenges and Lessons Learned when Measuring a Complex Intervention with Chart Review.

Challenge	Lesson Learned

Electronic chart spread across three software platforms	It is critical to gain access to the full chart in order to review all possible data.
Inconsistencies in the data	It is important to read through all available information, including the seemingly unimportant administrative details in the charts, to gain an accurate understanding of implementation factors of complex interventions.
Capturing the collaboration and flow of the implementation process	Allot additional time to complete data collection when measuring complex interventions with chart review.
Failing to see the forest through the trees	Chart reviewers must remain open to seeing and documenting new relevant data and patterns beyond what is recorded on the data collection form.

### Challenge 1: Data Extraction vs. Abstraction

The words “extraction” and “abstraction” are often used interchangeably when describing the medical chart review process, but their definitions illustrate a subtle but important difference that we found especially pertinent to measuring this complex intervention with this methodology. Extracted data are exact, word-for-word copies that can often be extracted automatically from the original information using software, while abstracted data are the important or general points that are usually manually recorded from the original information [38]. This nuance also illustrates the difference between using medical chart review for implementation research compared to traditional health services research or clinical research.

We found that a number of the transitional care actions we coded for required abstraction rather than extraction (this determination was only possible after data collection and could not have been made a priori). In fact, “Complete communication of information” was the only one transitional care action able to be captured completely through extraction because it is solely concerned with the information contained in the discharge summary. Discharge planning and patient education were amenable to both extraction and abstraction. For example, the presence of a discharge summary in the chart was an easily extractable data point and the existence of this summary indicates that discharge planning has occurred. However, to document the other actions involved in discharge planning, data had to be abstracted from providers’ notes. Data on all other transitional care actions were not amenable to extraction and were only amenable to the more nuanced and detailed abstraction method.

Table 3 summarizes which actions were amenable to extraction and abstraction, the features of each action that may be of interest in traditional research versus implementation research, and validation results based on provider interviews.

**Table 3:** Differences in Chart Review Methodology.

Transitional Care Action	Information was able to be extracted	Information needed to be abstracted	Features of interest in traditional research		Features of interest in implementation research		Chart data validated through provider interviews

Discharge Planning	Yes	Yes	•	Presence of Discharge Summary/Instructions	•	Who spoke to the patient or caregiver about discharge plans	Yes
Complete Communication of Information	Yes	No	•	Specific clinical information including diagnoses, medications, and test results are listed in Discharge Summary.	•	Who created, completed, and signed off on the Discharge Summary	NA
Availability, Timeliness, Clarity, and Organization of Information	No	Yes	•	Confirmation that Discharge Summary was electronically sent to follow-up providers	•	Who sent the Discharge Summary to the outside providers	Partially
					•	When did they send it	
Medication Safety	No	Yes	•	A Medication Administration Record is completed and available in chart	•	Who took the medication history or contributed information to it	Partially
					•	Who conducted medication reconciliation and how often did it occur	
Patient Education & Promotion of Self-Management	Yes	Yes	•	Confirmation that patient received counseling on discharge medications prior to discharge	•	Who provided the education	Yes
					•	Was it provided to patients and/or caregivers	
					•	When was it provided during the hospitalization	
Social and Community Supports	No	Yes	•	A Social Work or Case Management Consult Note is available in chart	•	Who provided this action	Yes
					•	What supports were recommended or used	
Advance Care Planning	No	Yes	•	A Palliative Care Consult Note is available in chart	•	Who put in the palliative care consult	Partially
					•	When was the consult put in	
					•	When was it completed	
Coordinating Care Among Team Members	No	Yes	•	Evidence that the patient's primary care physician was notified they were in the hospital	•	Who in the hospital communicated with providers outside of the hospital	Yes
					•	What providers outside of the hospital were contacted	
Monitoring and Managing Symptoms after Discharge	No	Yes	•	Confirmation that the patient received counseling on their follow-up care	•	Who provided the education	Yes
					•	Was it provided to patients and/or caregivers	
					•	When was it provided during the hospitalization	
Outpatient Follow-up	No	Yes	•	Scheduled follow-up appointment information appeared on the Discharge Summary	•	Who provided this action	Yes
					•	When were the outpatient follow-up appointments made during the hospitalization	
					•	Who were the appointments made with	

We believe that researchers setting out to use chart review for implementation research should be aware of the difference between extraction and abstraction. This distinction has been shown to affect the validity of chart review with extracted data because it can significantly underestimate the delivery of services due to providers often documenting the services they provide in non-structured fields such as free-text fields (which are not amenable to extraction) [39]. Therefore, in measuring the implementation process of complex interventions, abstraction rather than extraction may mitigate some of the limitations investigators have noted with chart review methodology because it will allow for a more comprehensive and nuanced review that captures the complexity of an intervention. It should also be noted, however, that with abstraction comes the introduction of possibly more variation because different coders may discern different patterns and record different levels of detail. This limitation can be mitigated through reliability testing prior to data collection and extensive recommendations on interrater reliability testing for qualitative content analysis exist [40,41,42,43,44].

### Challenge 2: Capturing the collaboration and flow of the implementation process

The second challenge we encountered was identifying and capturing collaboration between hospital providers and the flow of the implementation process. First, understanding and capturing how providers worked together to deliver care was not always obvious. For example, many of the “notes” or forms in the chart are authored by a single provider. A “Social Work Assessment” was solely completed by a social worker and a “Case Management Note” captured a single episode of care provided by a single case manager. This may lead one to make the assumption that the providers are working in silos. However, by taking the time to read through these notes we gained a much clearer picture of how the providers are working together.

For example, we saw a pattern emerge in the chart that suggested that case managers were evaluating every patient within 24 hours of admission and then social workers were often – but not always – evaluating patients after that. A further inspection of both the case managers’ and social workers’ documentation revealed that they were working together to provide discharge planning to patients. The case manager would determine if there was a need to involve social work (common reasons included the patient being likely to discharge to a facility or the patient needing advanced directives and power of attorney assistance) and then the case manager would initiate a referral to social work. A social worker would receive the referral and then evaluate the patient. The referral was not separately documented in the chart. It was only through reading the text in these providers’ notes that we observed evidence of this collaboration and process. Both the case manager and social workers confirmed this collaboration and process in our post-analysis interviews, thus highlighting the importance of these interviews as a validity check.

It was also challenging to identify the order and flow in which implementation occurred. While medical records offer rich, detailed information related to implementation such as timestamps and providers’ credentials that are often automatically recorded, the sheer amount of this data and its automatic creation can cause confusion when trying to follow the care delivery process. For example, many notes have timestamps of when the author creates/writes or updates a note. All notes listed in the EMR system are in chronological order based on their most recent timestamp. In most cases, providers will create new notes instead of going back into existing notes to document changes; therefore the chronological order in which notes appeared in the EMR is a good indication of the order in which providers document/provide care. However, there are times when providers update existing notes, thus changing the timestamp on the note in the EMR and subsequently changing the order in which the notes appeared in the EMR. This nuance was important for us to learn when trying to understand the order in which implementation processes occurred.

In one patient’s case, the patient was originally supposed to be discharged to a nursing facility but was later deemed stable enough to be discharged back home with their caregiver. This change was clear based on multiple providers’ notes in the EMR so we were confident when this change occurred; however, we discovered a later note in the EMR from a case manager discussing the patients’ pending nursing home transfer. At first we thought the case manager had the wrong information or may be confused but upon further inspection of the timestamp of the case manager’s signature on her note, we discovered that the information in that note was actually entered prior to the change in discharge plans. The note was updated later with minor changes so a new, more recent timestamp was given to the note, ultimately placing it out of chronological order in the EMR. We would have mistakenly assumed an error on the case manager’s part had we not read the note and paid close attention to the information that was automatically entered into the note, such as the timestamp.

It was also sometimes challenging to identify the features of implementation including who was delivering the intervention and when. For example, copies of patients’ discharge paperwork were found in one part of the medical record. Reviewing this paperwork gave us numerous data points including the mere presence or absence of the paperwork, the clinical information included in it, and the providers who completed and signed the paperwork (e.g., a physician and a nurse practitioner). However, upon further investigation, we could see in another part of the record that the discharge paperwork was actually created and populated with information by a registered nurse and then reviewed and “signed off” on by a physician and nurse practitioner. We were able to see how these different providers worked together for this one action because of the automated documentation in the part that logged the name, credentials, and timestamp of every action. Thus we were able to see, for example, that “Jane Doe, RN, 07-25-15, 14:25” created and updated the discharge paperwork in one part and then at a later date and time the paperwork was reviewed and signed by “John Doe, NP, 07-27-15, 08:12.”

Thus our second lesson learned is the importance of reading through all available information, including the seemingly unimportant administrative details in the charts. This practice facilitates a better understanding of an intervention’s implementation features.

### Challenge 3: Seeing the forest through the trees

The final challenge is less logistical than the previous ones but we feel it is perhaps more important. When conducting a chart review to abstract a complex intervention including its implementation features from the data, it is critical to remember to “see the forest through the trees.” In other words, it is important to remember that you are attempting to gain insight into a complex intervention *and* its implementation features, not pinpoint specific data points that you can quantitatively analyze. Chart reviewers must remain open to seeing and documenting new relevant data and patterns beyond what is recorded in the pre-determined data collection plan.

It is especially useful to have training in qualitative methods, especially content analysis, before embarking on a chart review of this nature. Qualitative methodology often allows for the results to emerge from the data without preconception rather than collecting data that will create a dataset to analyze for specific answers. This concept is useful for conducting a chart review in implementation research because it allows the researcher to gather a more holistic and rich picture of an intervention’s implementation features.

That is not to say that operationalizing specific data points is not important as noted earlier, we spent a great deal of time on this. But the importance of balancing focused, well-defined data collection with broad aims when conducting this type of chart review is the most important lesson we learned.

Additionally, allowing for both inductive and deductive data analysis is an important methodological step when conducting chart review for implementation research purposes. Many researchers are familiar with “top down” deductive methods in which theory is used to guide the research questions and hypotheses and then that theory is tested with data analysis 48. In comparison, “bottom up” inductive methods begin by examining specific existing patterns or events then through analysis identify larger phenomena and relationships [45].

### Recommendations for Researchers

Based on our experience conducting this study, we have compiled a list (Table 4 below) of recommendations for implementation researchers seeking to use this methodology. This list is not meant to be an exclusive or exhaustive list of recommendations for chart review methodology as many evidence-based, established guidelines already exist [3,4,7,13,14]. This list includes the recommendations that we believe are unique to implementation research that are absent from existing guidelines. We have divided these recommendations into three categories: pre-data collection, during data collection, and post-data collection.

**Table 4:** Recommendations for Conducting Chart Review in Implementation Research.

Timing	Recommendation	Details

Pre-data collection	Operationalize variables to measure process.	Create operational definitions of not only the intervention but the implementation process you are seeking to capture. This process should be done with people who have expertise in the intervention as well as people with expertise in implementation processes. This process should be iterative until consensus is reached on accepted definitions.
	Engage with current providers	Gather input from those believed to be currently delivering the intervention and entering data into the medical chart including input on what processes to code and where the processes are documented if they are at all.
	Pilot test data collection instrument	Pilot testing data collection instruments is a common and critical step when conducting any chart review. Our recommendation relates instead specifically to the number of charts to include and how to adapt the instrument based on the pilot test. Because we recommend not setting a sample size a priori and using a qualitative sampling approach, we recommend a similar approach to the pilot test and recommend reviewing enough charts until the coders feel comfortable with the instrument. Additionally, some have recommended that if a variable does not show up in more than 10% of the pilot tested charts then it should be cut from the data collection instrument [11]. We disagree with this for the purpose of implementation research where we are interested in the process and do not need a processes to be common for it to be of interest.
During data collection	Utilize free-text fields often	Allow for patterns or themes to emerge from the data that were not anticipated and have free-text fields incorporated into your data collection instrument in order to abstract these patterns or themes.
	Allow for wide variation in the time it takes to review a given chart	When planning the timeline for a study, allow for wide variation in the amount of time it will take to review an average chart and allot for additional time than one might first except in the study’s timeline.
Post-data collection	Engage with current providers	After preliminary data analysis has been conducted, we recommend reporting the results back to the providers you interviewed prior to data collection. In these interviews, we recommend asking the providers to confirm or deny the results of the chart review not only for their own roles but the roles of other providers i.e., ask a social worker if what appears in the chart to be a case manager’s role is seen in routine practice. We also recommend you ask providers their thoughts on the patterns you found in the chart data as this yielded rich context to the results of our study.
	Report methodology	This recommendation is again not unique using chart review for implementation but we want to reinforce the importance of reporting your methodology when publishing your study results. There are numerous existing reporting guidelines but we recommend that in addition to these you also report the steps outlined in this manuscript that you took specific to your implementation research study. This step is critical both in terms of transparency and replication efforts.

## Limitations and Considerations

Medical chart review is a commonly used methodology in fields such as epidemiology and clinical research [3]. There are many reasons investigators use chart review methodology, including convenience, the ability to collect data from a large sample, and in certain circumstances that depend on the pay grade of the data abstractor and number of charts, its relatively low cost [8,9,10,11]. There are of course limitations that still exist with this method regardless of the field in which it is used. The data abstracted from the chart is still reliant upon providers documenting their activities. In our case, for analysis, if there was no documentation in the chart of a transitional care action then we did not count that action as being completed. However, we believe it is critical that future implementation researchers using this method conduct follow-up interviews with providers after data analysis to understand if and how the documentation in the chart differs from what is actually done in practice. The results of these interviews should be reported in an effort to provide full disclosure on the accuracy of the chart data and provide context to the results of the chart data analysis.

An additional limitation of our study is that it was conducted at a single hospital, thus the results cannot be widely generalized. More studies are needed that look at the applicability of this methodology across multiple hospitals, outpatient settings, and different EMR systems. Our study was also conducted with only one data abstractor (BP) and future studies should use multiple data abstractors in order to increase the reliability and validity of the results. Despite these limitations, we feel confident that our results accurately depict the implementation of transitional care by hospital providers, a feeling shared by the hospital providers themselves.

Another important consideration in chart review is that what a provider believes they do in practice may still not accurately reflect what truly happens [12,46], thus increasing the uncertainty of which is more accurate: chart data or provider-reported data. Providers may subconsciously misrepresent their roles because they have an existing concept or idea about what their roles should be and are motivated to confirm this due to the self-evaluation process. The self-evaluation process is one in which an individual negotiates and modifies their self-concept (in this case, the idea of what the provider believes his or her role should be) based on motives including self-enhancement (making oneself appear better than they are) or self-verification (the need to verify what one thinks or him or herself of what others think of him or her) [39]. For example, if a social worker believes their role is discharge planning, they may report that they provide discharge planning to patients whether or not they do much or any discharge planning. Then when confronted with chart review results that reveal little documentation of discharge planning by social workers, they may report that they do in fact provide discharge planning to patients but that they simply do not document it because they, subconsciously, want to appear to be discharge planners because they think that will make them look better or they believe others see them as discharge planners and they want to conform this belief.

In our study, however, if this process did lead to a misrepresentation of providers’ roles in our results, it may not be problematic in the context of implementation research. For example, if social workers see themselves as the primary discharge planners and they are presented with an intervention or implementation strategy where they are asked to take the lead on discharge planning, they will view that intervention or implementation strategy as acceptable whether or not they actually do the majority of the discharge planning. However, this holds the potential to have contrasting and problematic effects on the feasibility of the intervention, and therefore this issue should not be ignored when considering the implications of the results of chart review in implementation research.

## Conclusion

Measuring the implementation of complex interventions is a cornerstone in health services research and chart review remains a frequently used methodology for clinical research. We believe we have demonstrated the value in this methodology for this purpose. Through our study we learned numerous lessons that proved key to our success including gathering input from providers, going the extra step to gain access to the full EMR, and using inductive and deductive analysis. We believe there are numerous benefits to using this methodology for this purpose and with more use and refinement it could emerge as a valuable and widely used method in the field.

## Data Accessibility Statement

The data generated during this study are not publicly available due to patient and participant confidentiality considerations.

## Additional File

The additional file for this article can be found as follows:

10.5334/egems.230.s1 Supplemental File 1 Interview Guide.
