# Supplementary material for: Measuring the Delivery of Complex Interventions through Electronic Medical Records: Challenges and Lessons Learned
Source: EGEMS (Wash DC). 2018 May 25;6(1):10. doi: 10.5334/egems.230 (PMC6078114; doi:10.5334/egems.230)
Supplement: Supplemental File 1 — Interview Guide. [file egems-6-1-230-s1.pdf]

Supplemental File 1: Interview Guide

Verbal consent: *Thank you for taking the time to talk with me today, (insert provider's name). As you read in the consent form, the purpose of this study is to understand what providers do to help older adults with dementia prepare to discharge from the hospital. I have a series of questions to ask you but you are free to skip any question or end the interview at any time. This interview should last no longer than 30 minutes. A few months from now when I'm done with analysis and have the results, I will be contacting you again to share the results with you and discuss your thoughts on them. Do you have any questions about this process or the study? Do you agree to this interview?*

Introduction: *Ok, just to recap what we talked about earlier, this interview is about how you care for older adults with dementia or cognitive impairment while they're in the hospital. Please think of these questions with regards to older adults with cognitive impairment.*

Question 1: *How do you help patients and their caregivers prepare for discharge?*

Probes: *Tell me more about what that looks like.*

<u>Repeat back the activities the provider listed for verification.</u>

Question 2: *Is there anything else that you do?*

<u>Repeat back the activities the provider listed for verification.</u>

Instructions: *Ok, next I'm going to ask you about some other activities that you might perform and I want you to tell me which ones you do, either routinely or rarely.*

<u>Ask about all ITC framework actions using checklist not discussed previously. Ask about each action one-by-one with the following question and prompts.</u>

Question 3 examples: *Do you ever take a patient's medication history? Do you discuss hospice care with the patient or their caregivers, if hospice is appropriate?*

Probe: Can you tell me more about that?

<u>Repeat Q3 until all ITC framework actions have been discussed.</u>

Instructions: *Now for all of these activities, I want to ask a little bit about how you document that you did them.*

<u>Ask about all ITC framework actions and any additional actions mentioned during Q1 and Q2 one-by-one with the following questions and prompts.</u>

Question 4 examples: *Do you document that you took a patient's medication history?*

Question 5: *Where do you document that?*

Question 6: *What do you specifically write or say to indicate that is what you did?*

Closing: *Well, that's all of the questions I have for today. Do you have any questions or final thoughts? Thank you for your time.*

Ask provider if they have any further questions about the study. Thank them for their participation, and remind them that I will be contacting them again at the end of the study to get their feedback on the results. Remind them of my contact information.

Interview ITC Framework Checklist

**ITC Framework Action**	**Endorsed by Provider in Q1**	**Endorsed by Provider in Q2**	**Endorsed by Provider in Q3**
Discharge Planning:<br>Planning ahead for hospital discharge while the patient is still being treated in the hospital. Includes collaborating with the outpatient provider and taking the patient and caregiver's preferences for appointment scheduling into account.			
Complete Communication of Information:<br>The content that should be included in the discharge summaries and other means of information transfer from hospital to post-discharge care.			
Availability, Timeliness, Clarity, and Organization of Information:<br>The availability, timeliness, clarity, and organization of the information above ensure post-discharge providers can access and quickly understand the information before assuming care of the patient.			
Medication Safety:<br>(1) Taking an accurate medication history, (2) reconciling changes throughout hospitalization, and (3) communicating the reconciled medication regimen to patients and providers across transitions of care.			
Patient Education & Promotion of Self-Management:<br>Teaching patients and their caregivers about (1) the main hospital diagnoses and instructions for self-care, including (2) medication changes, (3) appointments, and (4) whom to contact if issues arise. Confirming comprehension of instructions through (5) assessment of delirium and dementia and (6) teach-back, and (7) providing educational materials that are appropriate to the patient and caregiver's level of health literacy and preferred language are important.			
Social and Community Supports:<br>Enlisting the help of these supports is crucial for assisting patients with household activities, meals, and other necessities during recovery.			
Advance Care Planning:<br>May begin in hospital or outpatient setting and involves establishing goals of care and health care proxies, as well as engaging with palliative or hospice care if appropriate.			
Coordinating Care Among Team Members:<br>Synchronizing efforts across settings and providers is vital as they coordinate information, assessments, and plans as a team.			
Monitoring and Managing Symptoms after Discharge:<br>Monitoring for new or worsening symptoms, medication side effects, discrepancies, or non-adherence, and other self-management challenges.			
Outpatient Follow-up:<br>Appropriate and prompt post-discharge appointments with providers who have a longitudinal relationship with the patient.			
